# Fluorescently Labeled Peptide Increases Identification of Degenerated Facial Nerve Branches during Surgery and Improves Functional Outcome

**DOI:** 10.1371/journal.pone.0119600

**Published:** 2015-03-09

**Authors:** Timon Hussain, Melina B. Mastrodimos, Sharat C. Raju, Heather L. Glasgow, Michael Whitney, Beth Friedman, Jeffrey D. Moore, David Kleinfeld, Paul Steinbach, Karen Messer, Minya Pu, Roger Y. Tsien, Quyen T. Nguyen

**Affiliations:** 1 Division of Head and Neck Surgery, University of California San Diego, La Jolla, California, United States of America; 2 Department of Pharmacology, University of California San Diego, La Jolla, California, United States of America; 3 Department of Physics, University of California San Diego, La Jolla, California, United States of America; 4 Section of Neurobiology, University of California San Diego, La Jolla, California, United States of America; 5 Howard Hughes Medical Institute, San Diego, California, United States of America; 6 Division of Biostatistics, Moores Cancer Center, University of California San Diego, La Jolla, California, United States of America; 7 Moores Cancer Center, University of California San Diego, La Jolla, California, United States of America; University of Sydney, AUSTRALIA

## Abstract

Nerve degeneration after transection injury decreases intraoperative visibility under white light (WL), complicating surgical repair. We show here that the use of fluorescently labeled nerve binding probe (F-NP41) can improve intraoperative visualization of chronically (up to 9 months) denervated nerves. In a mouse model for the repair of chronically denervated facial nerves, the intraoperative use of fluorescent labeling decreased time to nerve identification by 40% compared to surgeries performed under WL alone. Cumulative functional post-operative recovery was also significantly improved in the fluorescence guided group as determined by quantitatively tracking of the recovery of whisker movement at time intervals for 6 weeks post-repair. To our knowledge, this is the first description of an injectable probe that increases visibility of chronically denervated nerves during surgical repair in live animals. Future translation of this probe may improve functional outcome for patients with chronic denervation undergoing surgical repair.

## Introduction

Nerve injury causes severe disability to patients by restricting movement, sensation or both. Transection injury to the facial nerve from cancer resection, or trauma,[1–4] leads to unilateral paralysis of the facial muscles, restricting facial movement and resulting in functional impediments such as constant drooling and loss of the blink reflex. Surgical reconstruction of the nerve can re-establish function although the achievable degree depends on the anatomical localization of the injury as well as the time passed since the event, both of which are important in choosing the appropriate reconstructive approach.[5] Surgical strategies aiming to achieve recovery of facial function including fundamental motor control and the ability to show emotion require the intraoperative identification of nerve branches proximal and distal to the site of injury. Primary neuroraphy and interpositional grafting, in particular, demand tension-free re-attachment, often necessitating re-routing or mobilization of the nerve segments.[6] Intraoperatively, identification of nerve segments distal to the site of injury becomes increasingly challenging with increasing duration from injury. Currently distal segments can be detected by electromyographic (EMG) tracing for up to 72 hours after injury,[7] but thereafter, surgeons have to rely solely on white light visual identification of the progressively degenerating distal segments. Extensive “searching” for the nerve segments increases the risk of additional injury to surrounding healthy tissues including adjacent nerves, and also extends operating time. These increased risks, combined with the increased cost of added surgical time, highlight the need for tools which will allow for reliable intraoperative nerve identification after the onset of nerve degeneration. We previously reported improved intraoperative peripheral nerve imaging in mice with the fluorescently labeled nerve binding peptide NP41 (F-NP41).[8] F-NP41 can be systemically applied and selectively labels intact and recently transected facial nerves.[9] In this study, we analyzed fluorescent labeling with F-NP41 of distal branches of the facial nerve for up to 9 months following transection of the main trunk. To address the critical issue of whether increased visibility improves the outcome of surgical repair, we performed nerve repair surgeries 6 weeks after transection and compared time to intraoperative nerve identification and postoperative functional recovery with and without F-NP41.

## Results

### Fluorescent labeling of degenerated distal facial nerve branches

**Fig 1:**
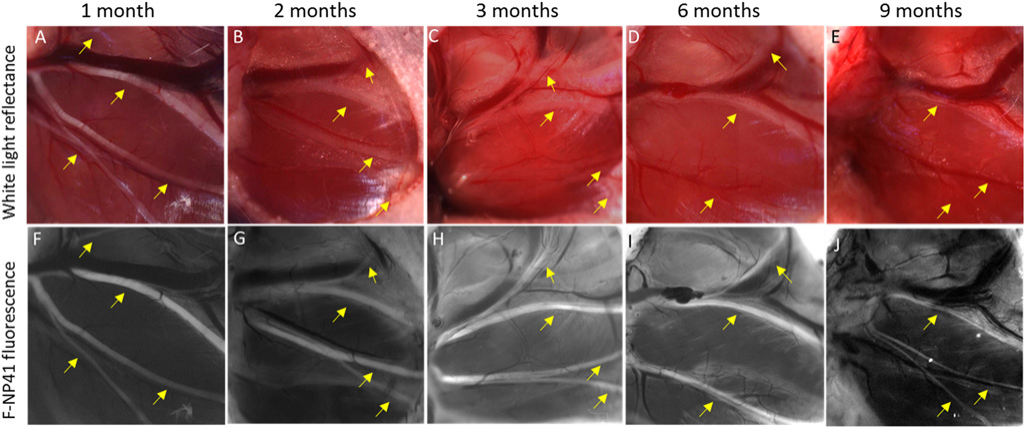
Improved visualization of degenerated facial nerve branches with F-NP41 labeling compared to white light reflectance alone. Yellow arrows in all images pointing to facial nerve branches. **A.** WL image of intact facial nerve branches. **B–E.** Facial nerve branches one, two, three and nine months after main branch transection. Nerve degeneration complicates visualization under WL. **F–J.** Corresponding fluorescence images obtained after systemic F-NP41 injection. Degenerated nerve branches are fluorescently labeled up to nine months after nerve transection.

**Fig 2:**
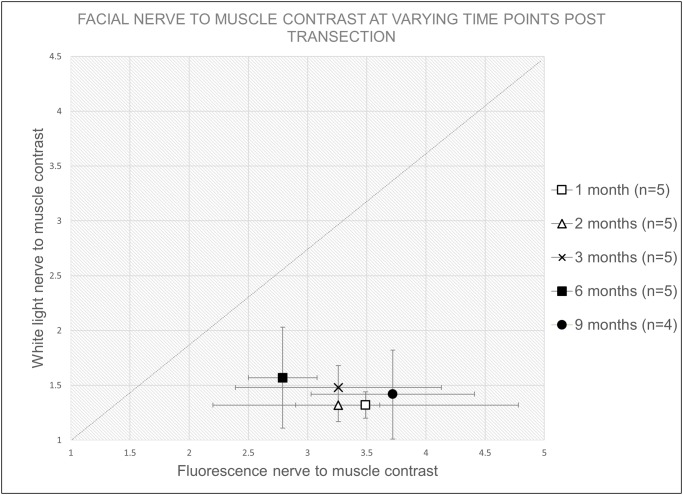
Plot of intensity ratios (mean and 95% CIs) showing improved visualization with F-NP41 labeling compared to white light reflectance of degenerated nerves at different time points after transection. Equal reflectance versus fluorescence is indicated by the dashed line (slope = 1). Values to the right of the line indicate an improved visualization with fluorescence imaging compared to white light, which was achieved at all measurement time points.

To determine whether previously reported F-NP41 could label chronically degenerated nerve branches, we unilaterally transected facial nerves in mice and imaged the distal branches at different time points after injury using F-NP41. To prevent spontaneous re-attachment of the proximal and distal segments after injury, a 2 mm section of the right facial nerve was surgically removed. Mice were allocated to one of five groups for terminal imaging 1, 2, 3, 6, or 9 months post-transection (n = 4–5 each). Animals from each group were imaged with both white light (WL) reflectance and fluorescence imaging. Prior to imaging, mice were anesthetized and administered F-NP41 intravenously at a dose of 15 nmols/g body weight. Following a washout period of 2.5 hours, facial nerve branches on the injured side were surgically exposed. Representative images comparing nerve contrast with WL versus fluorescence showed that the degenerative processes substantially affected nerve visibility under white light (Fig. 1). As early as one month after nerve transection, nerve to non-nerve tissue contrast under white light had decreased to near imperceptibility. In striking contrast, degenerated nerves could be easily visualized with F-NP41 dependent fluorescence as long as 9 months post transection. Quantitatively, the average intensity of distal nerve stumps, measured from fluorescent images following F-NP41 administration and normalized to non-nerve tissue, were between 78% and 164% greater when compared to intensity ratios measured from corresponding white light reflectance images, for all time points after nerve transection (Table 1, Fig. 2).

**Table 1:** Mean intensity ratios and the 95% confidence intervals of degenerated nerve to non-nerve tissue for white light and fluorescent images by time point.

Imaging Time Point After Nerve Transection	n	Intensity ratio (mean and 95% CI) of nerve to non-nerve tissue		
		White Light	F-NP41	% Difference in means
1 month	5	1.32 (1.20, 1.44)	3.49 (2.20, 4.78)	+ 164.4
2 months	5	1.32 (1.16, 1.48)	3.26 (2.91, 3.62)	+ 147.0
3 months	5	1.48 (1.28, 1.67)	3.26 (2.39, 4.13)	+ 120.3
6 months	5	1.57 (1.11, 2.03)	2.79 (2.50, 3.08)	+ 77.7
9 months	4	1.42 (1.02, 1.83)	3.72 (3.03, 4.41)	+ 162.0

Fluorescent labeling significantly increases nerve to background contrast up to 9 months after main branch transection. P-values comparing F-NP41 and WL were all <0.01 after Bonferroni adjustment.

**Fig 3:**
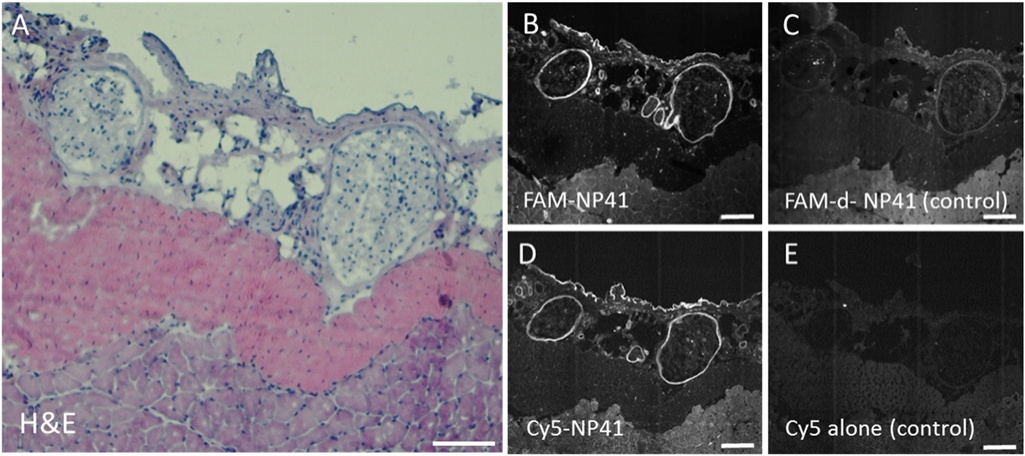
Confocal microscopic imaging of degenerated nerve sections after topical application of F-NP41 and control peptide. **A**. H&E stained sample of two degenerated facial nerve branches excised 10 weeks after main branch transection. **B.** Confocal fluorescence microscopy image after topical F-NP41 application of the same sample shows specific fluorescent labeling of the epineurium and perineurium of the degenerated nerve branches, while topical application of FAM-d-NP41 in **C**, serving as a control, shows no specific labeling. **D.** Section of the same tissue sample stained with Cy5-NP41 also shows fluorescent labeling of the degenerated branches, while application of free Cy5 in **E** show no specific labeling. Images B through E brightened 40%, scale bars 100 μm.

To confirm degeneration pathologically, nerve tissue from degenerated distal facial nerve branches was histologically analyzed from mice 6 weeks post transection. These transection nerves showed marked signs of Wallerian degeneration, including myelin degradation and macrophage infiltration, which lead to decreased visibility with WL reflectance (S1A, S1B Fig). Confocal microscopic imaging showed that topically applied F-NP41 and Cy5-NP41 label the perineurium/epineurium of degenerated peripheral nerves, rather than the progressively degenerating endoneurium, potentially explaining why fluorescence contrast is maintained post-degeneration (Fig. 3). The perineurium/epineurium has been shown to undergo scar formation upon transection,[10] but is not degraded, allowing for consistent NP41 binding even months after nerve transection.

### Improved intraoperative identification of facial nerve stumps with F-NP41

**Fig 4:**
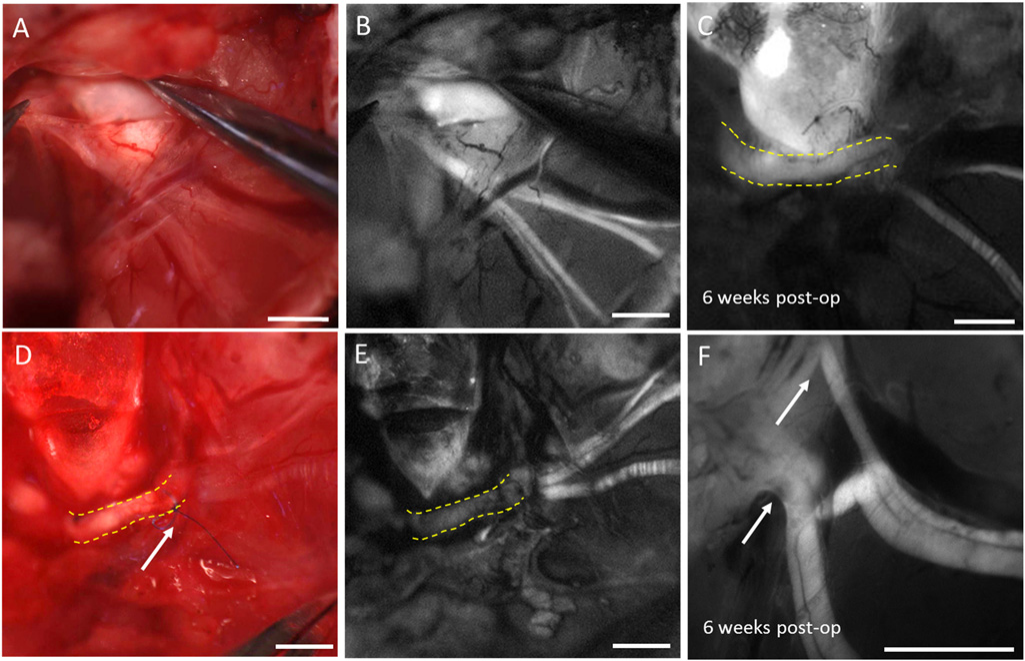
Intraoperative nerve visualization with fluorescence during repair surgery and to show postoperative nerve graft integration. Repair surgeries with a sciatic nerve graft were performed six weeks after nerve transection. Intraoperative fluorescence guidance improved visualization of the degenerated distal nerve stump (white light image **A** versus corresponding fluorescence image **B**). After nerve graft implantation (yellow stippled outline in corresponding WL and fluorescence images **D** and **E**) with epineural sutures (white arrow in d), the donor graft did not show any fluorescence signal. 6 weeks after the repair procedure, the graft (yellow stippled outline in **c**) showed fluorescence signal after systemic F-NP41 injection indicating graft acceptance and established blood supply. **F** shows a high magnification fluorescence image of the regenerating distal nerve branches sprouting into the nerve graft (white arrows). Scale bars in a, b, d, e: 1mm, in c, f: 0.3mm.

To assess the value of improved intraoperative visualization of chronically degenerated facial nerves for surgeons, nerve repair surgeries with a cable graft were performed 6 weeks after facial nerve transection injury and confirmation of nerve degeneration. In this study, mice were allocated to one of two groups: surgical repair performed with fluorescence guidance (n = 11) or surgical repair performed under white light alone (n = 8). In the fluorescence group, 15 nmols/gram body weight F-NP41 was administered intravenously 2.5 hours prior to the repair surgery. During the procedure, the proximal and distal stump of the facial nerve were imaged with fluorescence optical microscopy to aid nerve identification. Intraoperative fluorescent labeling aided visualization of the degenerated distal facial nerve stumps during the grafting procedure (Fig. 4), leading to a significantly decreased time to identification of the distal nerve stumps prior to graft attachment. Time to identification for the fluorescence (FL) group with F-NP41 was reduced by 39.4% compared to the white light reflectance (WL) control group (15.4 ± 3.8 min vs. 25.4 ± 3.2 min, Student’s t test, p<0.0001). Fluorescent labeling provided lasting contrast after the initial washout period, allowing visualization over the entire duration of the surgical procedure. Long-lasting specific labeling after a comparatively short wash-out period is particularly valuable for potential clinical applications, because complex nerve reconstruction surgeries in humans can take up to several hours.

### Functional nerve recovery after cable graft procedure

**Fig 5:**
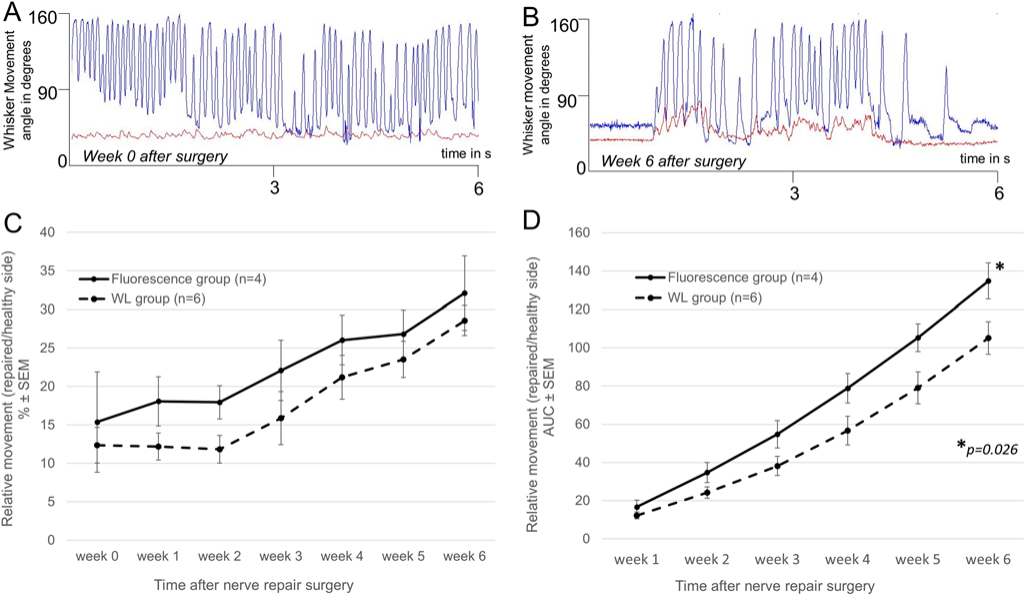
Assessment of functional recovery after facial nerve repair. **A.** Representative whisker movement amplitude plot of a repaired whisker immediately after surgery (red line) and the contralateral healthy whisker (blue line) obtained over a 6-second period of continuous whisking using automated whisker tracking technology. The repaired whisker shows only small fibrillations indicating complete denervation. **B.** Representative whisker movement amplitude plot of a repaired whisker 6 weeks after surgery (red line) and the contralateral healthy whisker (blue line) obtained over a 6-second period. The repaired whisker shows recovery of movement. **C**. Relative postoperative whisker movement amplitude, i.e. repaired side movement in % of healthy side movement. **D.** Cumulative functional improvement (area under curve—AUC) calculation showing there was faster functional recovery improvement in the FL (solid line) group compared to WL (stippled line). Student’s one- sided t test p value provided at the final time point.

To determine the degree of functional motor recovery after surgical facial nerve repair, we systematically tracked ipsilateral and contralateral whisker mobility at weekly intervals postoperatively for six weeks. Eight mice with surgically repaired facial nerves were excluded over the course of the follow-up period due to deterioration in health or lack of acclimatization to head restraint. Whisker movement amplitude is a common parameter to assess facial nerve function in mouse and rat models,[11, 12] because vibrissal pilorector muscles are primarily innvervated by the buccal branch of the facial nerve. Rodents acquire tactile information by rhythmically sweeping their vibrissae with extensive bilateral symmetry, therefore the contralateral whisker provides an immediate internal reference.[13–16] Six (6) mice from the WL group and five (5) mice from the FL group successfully completed the full experimental protocol of complete facial nerve transection followed by confirmation of functional loss 6 weeks post-transection and surgical nerve repair with a nerve graft, including intra-operative distal stump identification (with or without NP-41) and postoperative whisker tracking. Technically successful nerve repair was determined as an increase in absolute values of the ipsilateral whisker movement amplitude in degrees 6 weeks post repair (Fig. 5B) compared to values obtained immediately after surgical resection (Fig. 5A) One mouse from the FL group did not show any evidence of functional recovery at any time following surgery, possibly due to surgical error of repair and was therefore excluded from analysis (S2 Fig). For the recovery analysis, the relative movement amplitude of the repaired side whisker at the different time points was calculated, i.e. its movement amplitude in % of the corresponding whisker on the healthy contralateral side. Hereby inter-individual differences in full whisking potential were accounted for. At 6 weeks post transection and prior to repair, relative movement amplitude was similar between the WL reflectance group (mean ± SD: 12.4 ± 5.7%, n = 6) and the FL group (15.4 ± 13.1%, n = 4, p = 0.31). Mice from the FL group tended to have slightly higher functional recovery compared to mice from the WL group although this difference was not statistically significant (Fig. 5C). Average whisker movement amplitude at 6 weeks on the surgically repaired side compared to the contralateral healthy side was 32.1 ± 9.6% (FL group) and 28.6 ± 4.3% (WL group), p = 0.23. Because relative whisker movement amplitude after surgery tended to be slightly higher in the fluorescence guided surgery group compared to the WL group at all-time points, we compared the cumulative functional improvement (area under the curve—AUC) from week 0 to 6 between the two groups (Fig. 5D). The FL group had a progressively greater AUC beginning at week 2 and extending through week 6, and the 6 week cumulative improvement was significant (mean ± SD: 135 ± 19% for FL vs. 105 ± 21% for WL, p = 0.026), suggesting that improved intraoperative nerve stump identification with F-NP41 can improve the speed, if not necessarily the extent, of postoperative functional recovery. To confirm successful nerve fusion, the repair site was reopened and reimaged 6 weeks after surgical repair, using fluorescence imaging of the facial nerve after F-NP41 injection. Animals from both groups showed fluorescent labeling of the nerve graft indicating successful graft integration and established blood supply (Fig. 4C,F). Sections of distal facial nerve branches were excised showed signs of regeneration in our histological analysis (S1C Fig).

## Discussion

F-NP41 potentially provides surgeons with a unique tool to aid nerve repair surgeries. We show that degenerating facial nerve branches can be fluorescently labeled with F-NP41 up to 9 months after the main branch of the nerve has been transected, facilitating significantly improved intraoperative identification compared to white light reflectance alone. To our knowledge, this feature is unique among nerve identification technologies reported to date. Facial nerve surgical repair in patients are performed up to two years after injury and functional recovery is attainable after delayed surgeries with facial nerve interpositional grafts, hypoglossal-facial anastomosis or cross-facial nerve grafts.[17–22] However, re-identification of the degenerated distal branches poses a significant challenge. EMG monitoring, as well as recently reported novel nerve tracers, require intact axonal pathways, to successfully detect or label nerves.[23, 24] However, many components of the endoneurium begins to undergo Wallerian degeneration processes as early as 24h after nerve transection.[25, 26] Our analysis suggests that F-NP41 labels the perineurium/epineurium of nerves, which does not undergo the same degree of degeneration, and provides much improved contrast for degenerated nerve visualization compared to white light reflectance. Intraoperative fluorescence guidance significantly reduced the time necessary to identify the degenerated distal nerve branches during nerve repair surgeries in this study. In clinical practice, the reduction in time to nerve branch identification translates into decreased total time under anesthesia for patients, decreasing total operating time and costs. Furthermore, the benefit of faster and more reliable identification of distal degenerated nerve branches limits the risk of damaging surrounding tissue. Particularly in head and neck surgery, the anatomical density of important structures such as blood vessels and nerves demands utmost care and limiting invasiveness as much as possible.[27] We found that the accelerated intraoperative identification of the facial nerve stumps translated to a faster functional recovery in the FL compared to the WL group despite the relatively short post-operative follow-up period and small sample size. While nerve recovery in mice is much faster than in humans, it is important to note that complete functional recovery after nerve repair surgery is not attainable in mice, regardless of the repair approach, most likely due to a combination of excessive axonal branching which has been documented in rodents at the transection site as well as at the target muscles.[28–31] To avoid potential observer bias, we invested significant effort to evaluate functional recovery using automated whisker tracking and not by observation in order to derive an objective measure of recovery. We hypothesize that the decreased time to intraoperative nerve stump identification helped to reduce the local tissue trauma and associated effects, such as swelling and inflammation, to tissue surrounding the nerve, hereby facilitating the initial phase of recovery. This study is a “proof of concept” study demonstrating feasibility of highlighting chronically degenerated nerves during surgery. Novel intraoperative applications typically require a high degree of familiarity with the experimental setup, and training multiple surgeons before proof of concept is not feasible due to financial and animal care and use restrictions. More extensive investigations, potentially in larger animal models with multiple surgeons performing the procedures are required to confirm these promising findings. Future translation of F-NP41 can potentially improve functional outcome for repair in patients with chronic denervation.

## Materials and Methods

### Fluorescently labeled probe

NP41 was synthesized as previously described.[8] For the visualization of facial nerve branches after main branch transection, the fluorescent dye 5(6)-carboxyfluorescein (FAM) was coupled via its carboxyl group to the epsilon amino group of a C-terminal lysinamide, resulting in F-NP41 = Ac- SHSNTQTLAKAPEHTGK[5(6)FAM]-amide.

**Animals and overview of experiment.** 24 female SKH-1 nude albino wild-type mice and 19 female C57/Bl6 mice (Charles River, Wilmingham, MA) were used in this study. We used SKH mice for the intensity quantification experiments because surgeries and imaging could be performed without prior hair removal. For the nerve repair procedures and functional follow-up, C57/Bl6 mice were used with regard to potential follow-up experiments in which we plan to utilize the improved visualization of degenerated nerve pathways for the application of nerve growth stimulating factors. Ultimately, we would aim to use murine stem cells. Currently commercially available murine stem cells are generated from the C57/Bl6 strain which prompted us to use this strain for this initial proof-of-concept study. All animal studies were approved by the University of California, San Diego Institutional Animal Care and Use Committee (protocol number S05536). 11 of the C57/Bl6 mice were followed up after surgery to evaluate functional facial nerve recovery measured by recovery of whisker movement amplitude over a six week period.

### Nerve transection surgeries

The right facial nerve of mice weighing 25 to 30 g was transected as follows: Each animal was anesthetized with an intraperitoneal injection of ketamine (150 mg/kg) and xylazine (10 mg/kg). A 1.5-cm lateral skin incision was made inferior to the external auditory canal (EAC) along the course of the facial nerve. The parotid gland was dissected off the underlying branches of the facial nerve as needed. The main trunk of the facial nerve was followed as it wrapped around the inferior border of the EAC and anterior to the posterior belly of the digastric muscle. Under microscopic visualization, the main trunk of the facial nerve inferior to the EAC was atraumatically released from the underlying tissue and a 2 mm piece was excised with microscissors. Excision prevented spontaneous reattachment of the proximal and distal stump. The incision was closed in a single layer.

### Fluorescent labeling and intraoperative imaging of degenerated facial nerve branches

24 SKH-1 mice which had undergone the nerve transection procedure were allocated to one of 5 groups: White light (WL) reflectance and fluorescent imaging 1, 2, 3, 6, or 9 months post facial nerve transection (n = 5 each, except last group n = 4). On the day of imaging, following anesthesia, F-NP41 was administered intravenously at a dose of 15 nmols/g body weight. Following a washout period of 2.5 hours and repeat anesthesia, facial nerve branches on the injured side were surgically exposed and imaged. For this purpose, a 2.5 cm lateral skin incision was made along the course of the three distal branches of the facial nerve and the facial skin was retracted. Using a customized Olympus fluorescence dissecting microscope, first, the distal nerve branches were imaged with white light reflectance. Thereafter, the nerve branches were imaged with fluorescence. Peptide fluorescence was imaged with LED (450–490nm excitation and 500–550 nm emission). Videos were acquired for subsequent signal to background ratio analysis.

### Analysis of signal-to-background ratio

Using images acquired as described above, nerves and adjacent non-nerve tissue were hand-selected using the arbitrary shape tool in Image J. The mean pixel intensities within the selected areas were compared for individual nerve branches against adjacent muscle tissue. Two nerve branches were quantified per mouse and three regions of interest (ROI) were selected per branch in the field of view. Exactly the same ROIs were evaluated on corresponding fluorescence and WL images. Results of signal-to-background ratios for reflectance and fluorescence were compared and plotted for each time point.

### Surgical nerve repair

The right facial nerves of 19 C57/Bl6 mice were transected as described above. 11 mice with confirmed functional defect 6 weeks post-transection were used for surgical repair. Nerves were repaired 6 weeks after initial transection by a cable grafting procedure. Surgical nerve repair for both groups were performed as follows: Under anesthesia, hair on the right side of the face was removed using depilatory cream. A 2 cm incision was then made under the EAC and meticulous dissection was performed to identify the proximal and distal stumps of the previously transected facial nerve main branch. In the fluorescence guided group, the proximal and distal facial nerve stump were repeatedly imaged with fluorescent optical microscopy to aid nerve identification. The extent of the skin incision was expanded if necessary. At the same time, a second surgeon harvested a 3–4 mm sciatic nerve branch graft from a C57/Bl6 mouse serving as donor. One donor mouse provided nerve grafts for two surgical repair procedures since the right and left sciatic nerves were both utilized as grafts. Donor mice were sacrificed immediately prior to the harvesting procedure. The harvested nerve grafts were sutured to the epineurium of the proximal and distal stumps of the transected facial nerves with 10–0 nylon sutures (Ethicon, San Angelo, TX). The skin incision was closed and the animals were returned to their pre-heated cages to recover from the procedure. The time necessary to identify the proximal and distal nerve stumps after skin incision was quantified for every mouse in both groups.

### Functional assessment

Nerve function was assessed with whisker tracking over a six week period after surgical nerve repair. Measurements of whisker movement were performed weekly after nerve repair surgery to assess regeneration of nerve function. To allow the mice to recover sufficiently from anesthesia, the first measurement (week 0) was performed at day 3 following surgery. Thereafter, measurements were performed at weekly intervals for 6 weeks. Mice which showed any signs of discomfort over the course of the follow-up period or which could not be acclimatized to head restraint during recording sessions were excluded from the functional recovery analysis. A daily assessment of animal health was performed by animal care personnel not involved in the study. The change in whisker movement amplitude (defined as the difference between maximal retraction and protraction in degrees) of one large vibrissa on the side of the injured facial nerve was compared to the equivalent vibrissa on the contralateral healthy side over time after nerve repair surgery. To measure whisking, vibrissa motion was recorded in awake non-sedated head-fixed mice, a method which has been previously described.[13, 32–35] To enable head fixation, prior to the nerve repair surgeries, a brief surgical procedure was performed under anesthesia during which a shortened L-shaped micro hex key (0.7 mm diameter, Eklind Tools, Franklin Park, IL) was attached to the skull bone of the mice with dental cement (C&B Quick Cement Cystem, Parkell Inc., Edgewood, NY). A 1.0 cm skin incision was made along the midline of the skull after hair removal with depilatory cream. After attachment of the device, the wound was closed, with a 6–0 non-resorbable suture (Ethicon, San Angelo, TX) covering the short end of the L-shaped key. The long end protruded through the skin incision at a 90 degree angle. For measurements of whisker movement, mice were gradually acclimatized to head restraint and immobilization in a plastic tube (*Tailveiner* restrainer, Braintree Scientific, Braintree, MA). During the recording session, mice were head fixed using a custom-made head restraining mount which attached to the previously implanted hex key. All but two corresponding whiskers, one on each side, were trimmed down to the skin. Vibrissa motion was monitored with a Basler 602f camera (Basler Inc., Exton, PA). 360x250 pixel planar images were acquired at 250 Hz with a white light emitting backlight for trials of 10s each. After video acquisition, vibrissa angle was automatically tracked using custom-written MATLAB software by fitting a line to the spatially contiguous pixels comprising the initial 5 mm segment of the vibrissa base. Nerve repair was considered technically successful only if there was an increase in absolute ipsilateral whisker movement amplitude in degrees over the course of the 6-week follow-up period. Technically successful nerve repair was considered a requirement for inclusion in the analysis comparing fluorescence guided surgery to white light surgery.

### Histology

To histologically assess the degree of Wallerian degeneration at the time of nerve repair surgery, as well as the degree of postoperative recovery, tissue samples of distal nerve branches were excised and frozen in optimum tissue cutting (OCT) formulation (Tissue Tek, Thermo Fisher Scientific, Waltham, MA) after the nerve imaging procedures described above. Histological analysis was performed after staining with luxol fast blue and cresyl violet on samples of healthy facial nerve branches, facial nerve branches six weeks after transection of the main branch of the nerve and samples collected from mice six weeks after the nerve reconstruction procedure.

### Confocal fluorescence microscopy of denervated nerve sections

To further characterize the localization of F-NP41 binding to degenerated nerves, F-NP41 and Cy5-NP41 were topically applied to cryosections (10μm) of a degenerated facial nerve sample excised 10 weeks after main branch transection. FAM-d-amino acid-NP41 and Cy5 free-acid served as controls and an additional slide of the same tissue sample was stained with hematoxylin and eosin (H&E) to confirm the presence of nerve tissue. Fluorescently stained samples were imaged with confocal fluorescence microscopy (Zeiss, Fluorescein: 488 ex/505 longpass em; Cy5: 632 ex/650 longpass em).

### Statistical analysis

Nerve to non-nerve contrast ratios were calculated for each ROI. For each mouse, a summary contrast ratio was obtained by averaging the ratios over all the six ROIs (three ROIs on two nerve branches). For each time point, a two-sided paired t-test was used to compare these summary ratios between the two groups (WL vs. FL). Bonferroni correction was made for multiple testing adjustment. In general, a two-sided Welch’s t-test was used to compare between the two groups for a continuous outcome such as time to intraoperative identification of facial nerve stumps. Functional nerve repair outcome of the whisker movement amplitude was first summarized using area under the curve (AUC) for each subject, and was then compared between the two groups (WL vs. FL) using a 1-sided Welch’s t-test. A one-sided t-test was used here because it was hypothesized that the FL group was to be associated with a better functional outcome due to the fact that FL uses much less time to nerve identification during surgical repair which results in reduction of local tissue trauma.

## Supporting Information

S1 Fig Histological analysis of facial nerve samples. **A.** Luxol fast blue (stains myelin, light blue color) and cresyl violet (nuclei) staining of samples of a healthy distal facial nerve branch. **B.** Section excised six weeks after main branch transection shows marked signs of Wallerian degeneration, including degradation of the myelin sheath, higher Schwann cell density and macrophage infiltration (white arrows). **C.** Distal facial nerve branch excised 6 weeks after repair surgery with a nerve graft shows increased myelin and reduced Schwann cell density compared to **B**, indicating the onset of nerve regeneration. Scale bars: 100 μm.
(TIF)

S2 Fig Plot showing the absolute postoperative whisker movement amplitude of every mouse from the FL group (solid lines, n = 5) and from the WL group (stippled lines, n = 6) in degrees. The difference in whisker amplitude from week 0 to week 6 following surgical repair is noted at the end of each line. A positive value indicates successful surgical repair and return of function. A negative value or zero indicates failure of surgical repair leading to lack of return of function. All mice with positive values were included in the analysis. One mouse from the FL group (red line) had a value of -1 and was excluded from analysis.
(TIF)
