# Supplementary figures and images for: Fluorescently Labeled Peptide Increases Identification of Degenerated Facial Nerve Branches during Surgery and Improves Functional Outcome
Source: PLoS One. 2015 Mar 9;10(3):e0119600. doi: 10.1371/journal.pone.0119600 (PMC4353702; doi:10.1371/journal.pone.0119600)

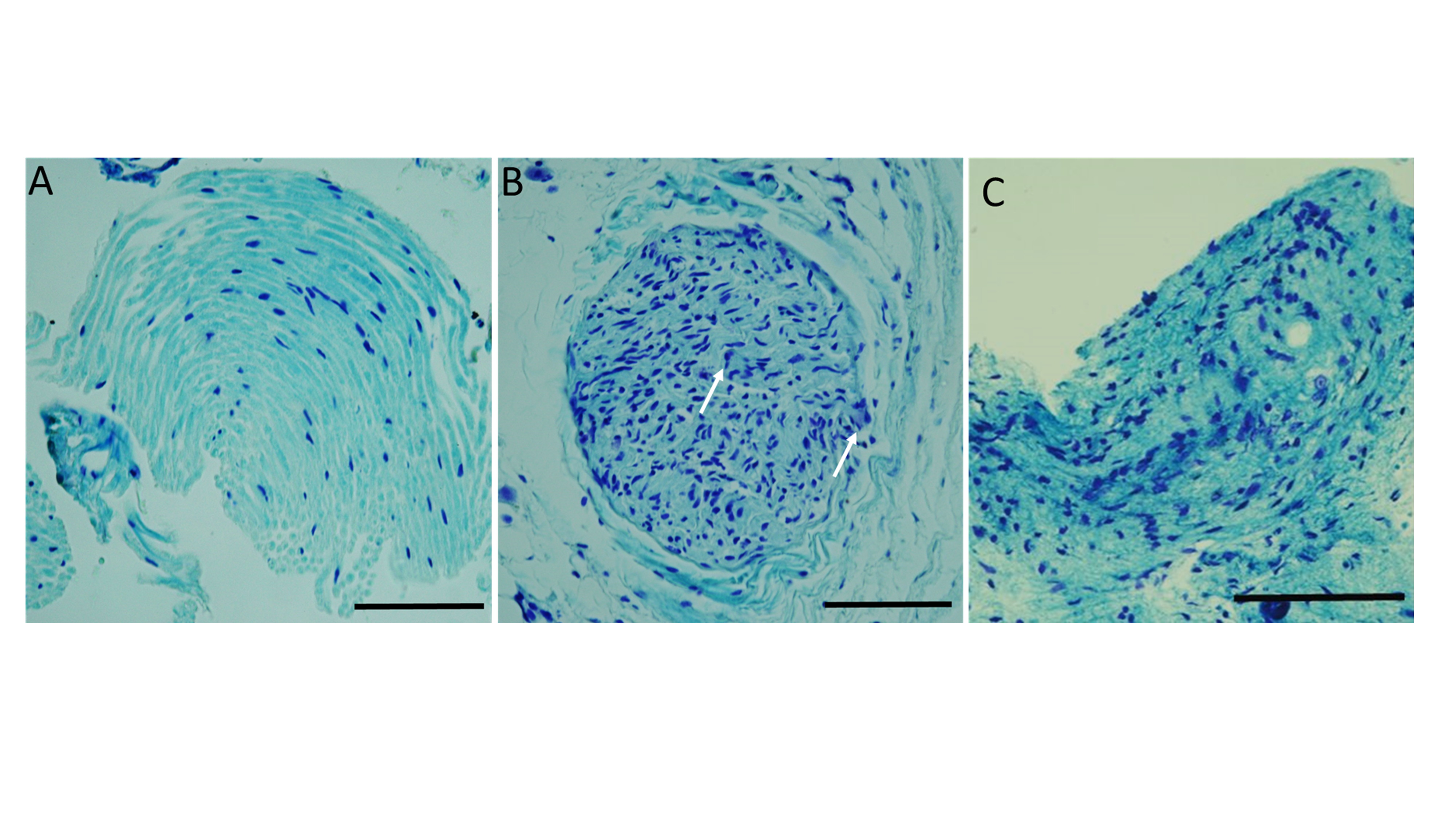

Supplement: S1 Fig — A. Luxol fast blue (stains myelin, light blue color) and cresyl violet (nuclei) staining of samples of a healthy distal facial nerve branch. B. Section excised six weeks after main branch transection shows marked signs of Wallerian degeneration, including degradation of the myelin sheath, higher Schwann cell density and macrophage infiltration (white arrows). C. Distal facial nerve branch excised 6 weeks after repair surgery with a nerve graft shows increased myelin and reduced Schwann cell density compared to B, indicating the onset of nerve regeneration. Scale bars: 100 μm. (TIF) [file pone.0119600.s001.tif]

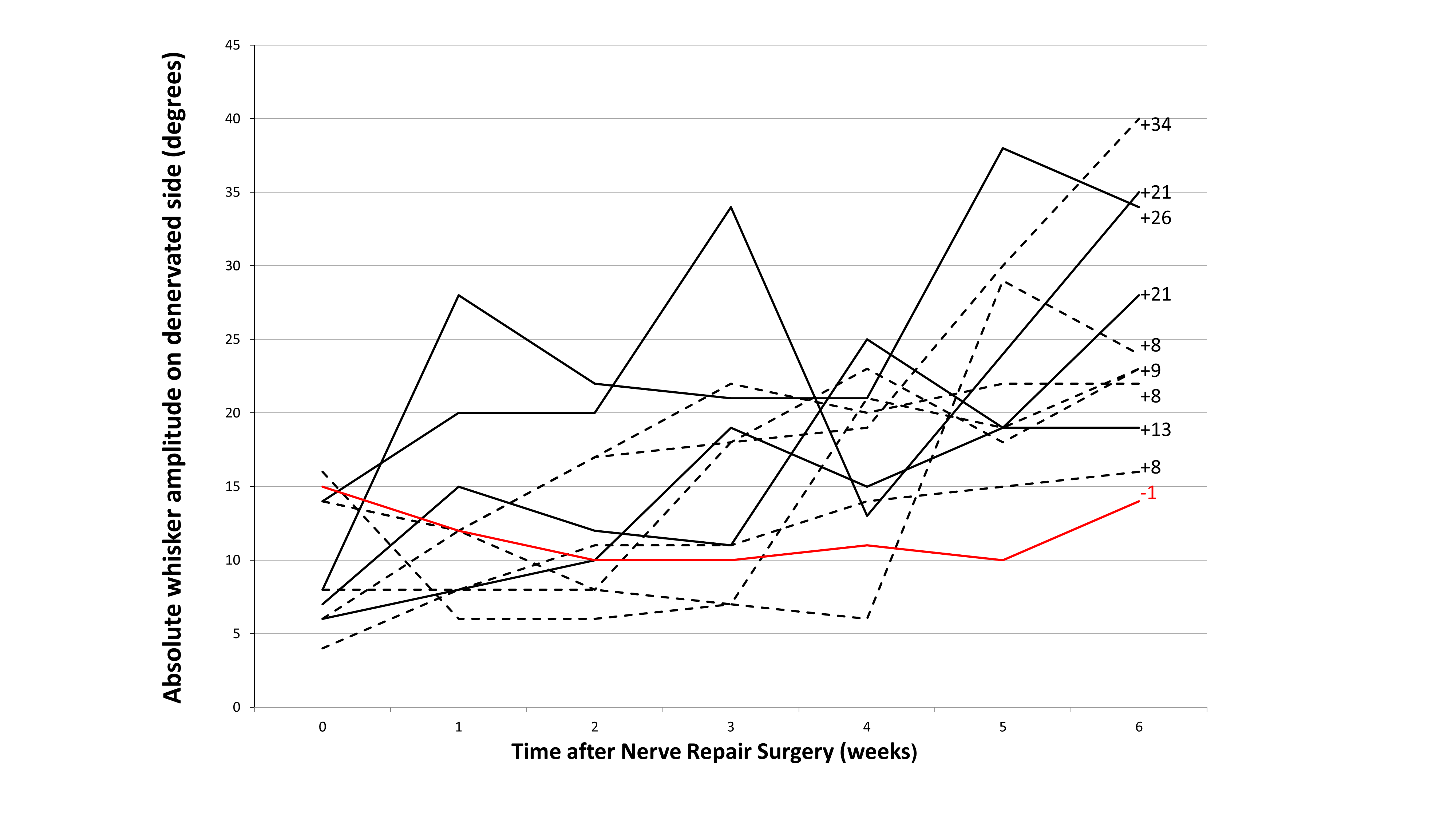

Supplement: S2 Fig — The difference in whisker amplitude from week 0 to week 6 following surgical repair is noted at the end of each line. A positive value indicates successful surgical repair and return of function. A negative value or zero indicates failure of surgical repair leading to lack of return of function. All mice with positive values were included in the analysis. One mouse from the FL group (red line) had a value of -1 and was excluded from analysis. (TIF) [file pone.0119600.s002.tif]
